# Draft Genome Sequence of Novel Staphylococcus epidermidis Strain EVL2000, Exhibiting Pathogenicity against Caenorhabditis elegans

**DOI:** 10.1128/mra.01239-21

**Published:** 2022-03-14

**Authors:** Nathan Do, Brian D. Ackley, Patrick Lansdon

**Affiliations:** a Department of Molecular Biosciences, University of Kansas, Lawrence, Kansas, USA; University of Rochester School of Medicine and Dentistry

## Abstract

Staphylococcus epidermidis is a frequent cause of nosocomial infections occurring after the insertion of indwelling medical devices. Here, we report the 2.5-Mb draft genome of S. epidermidis strain EVL2000, which was identified during an examination of nematode susceptibility to microbial pathogens.

## ANNOUNCEMENT

Long considered a benign colonizer of skin and mucous membranes, Staphylococcus epidermidis is now one of the most frequent causes of nosocomial infections during the insertion of indwelling medical devices (1–6). S. epidermidis strains exhibit significant genomic diversity regarding virulence and host modulatory factors, allowing isolates to adapt to and persist in their ecological niches (7, 8). Thus, whole-genome sequencing, assembly, and annotation of novel S. epidermidis strains will improve our understanding of S. epidermidis as both a pathogen and a member of the microflora.

While examining Caenorhabditis elegans susceptibility to microbial pathogens, we serendipitously cultured a bacterial contaminant from nematode growth medium (NGM) plates that exhibited remarkable differences in nematocidal activity in two genetically diverged C. elegans wild isolates (9). The strain was streaked on a Luria-Bertani (LB) agar plate and incubated at 37°C for 24 h. A single colony was grown in low-salt LB medium at 37°C for 24 h with gentle shaking. DNA was extracted using a QIAquick miniprep column (Qiagen). Universal primers 27F and 1492R were used to amplify the 16S rRNA region, and the resulting Sanger sequencing product was aligned to Staphylococcus epidermidis. We provided the strain with the designation EVL2000.

For whole-genome sequencing, DNA was extracted from liquid culture using a DNeasy blood and tissue kit (Qiagen) following a modified manufacturer’s protocol for Gram-positive microorganisms, with increases in lysozyme concentration (40 mg/mL) and incubation times for lysozyme (2 h) and proteinase K (3 h). The quantity and quality of extracted DNA were assessed using a Qubit 2.0 fluorometer (Invitrogen) and an Agilent TapeStation 2200 (Agilent), respectively. Library preparation and sequencing were performed by the Genome Sequencing Core at the University of Kansas (KU). The DNA library was prepared using Illumina Nextera chemistry following the manufacturer’s instructions and was sequenced on the Illumina MiSeq platform with the Illumina v2 reagent kit using the paired-end protocol (2 × 150 bp). Sequencing yielded 1,398,550 reads with 85× coverage.

Reads were quality checked with FastQC v0.11.9 (10). Trimming was performed using fastp v0.20.0 with the --correction flag enabled to remove Illumina adapter sequences and low-quality reads with Phred scores of <30 (11). Trimmed reads were *de novo* assembled using SPAdes v3.13.1 with kmers set to 21, 33, 55, and 77 and the --only-assembler and --careful flags enabled (12). Contigs of <200 bp were removed, and QUAST v5.0.2 was used to evaluate the genome assembly (13). The assembled genome contained 100 contigs (>200 bp), was 2,509,527 bp in size, and had a GC content of 31.96% and an *N*_50_ value of 105,624 bp. Annotation of the S. epidermidis EVL2000 genome assembly using the NCBI Prokaryotic Genome Annotation Pipeline (PGAP) v6.0 identified 2,449 genes, with 2,313 protein-coding sequences, 68 pseudogenes, and 68 RNAs (7 rRNAs, 57 tRNAs, and 4 noncoding RNAs) (14).

### Data availability.

The whole-genome sequence of S. epidermidis EVL2000 has been deposited in DDBJ/ENA/GenBank under the accession number JAJSYT000000000. The version described in this paper is JAJSYT010000000. Raw reads have been deposited in the Sequence Read Archive (SRA) under the accession number SRR17282514.
